# Dynamics of a Sporadic Nosocomial *Acinetobacter calcoaceticus – Acinetobacter baumannii* Complex Population

**DOI:** 10.3389/fmicb.2019.00593

**Published:** 2019-03-22

**Authors:** Pilar Villalón, Montserrat Ortega, Juan A. Sáez-Nieto, Gema Carrasco, María J. Medina-Pascual, Noelia Garrido, Sylvia Valdezate

**Affiliations:** Laboratorio de Referencia e Investigación en Taxonomía, Centro Nacional de Microbiología, Instituto de Salud Carlos III, Madrid, Spain

**Keywords:** *Acinetobacter calcoaceticus-baumannii* complex, sporadic strains, epidemic strains, species identification, clonal distribution, antimicrobial susceptibility

## Abstract

Our objective was to improve current knowledge of sporadic (Spo) nosocomial *Acinetobacter* *calcoaceticus-Acinetobacter baumannii* (Acb) complex populations, and thus better understand the epidemiology of Spo and endemoepidemic (EE) strains. Between 1999 and 2010, 133 isolates of Spo Acb complex were obtained from a single hospital. Species were identified by *gyr*B-PCR, and via *gyr*B- and *rpo*B-sequencing. Clonal analysis was undertaken using pulsed-field gel electrophoresis (PFGE) and multilocus sequence typing. Susceptibility to antimicrobial agents was determined by microdilution and E-tests. Carbapenemase genes were detected by PCR. One hundred and one PFGE types were detected. *A. baumannii* was the most common (67/101 PFGE types), followed by *Acinetobacter pittii* (22/101), *Acinetobacter lactucae* (6/101), and *Acinetobacter calcoaceticus* (2/101). *gyr*B, *rpo*B1, and *rpo*B2 sequencing returned 49, 13, and 16 novel sequences, respectively. Sixty-three sequence types (STs) (38 new STs and 66 new alleles) were detected; the most common were ST2 (29/133 isolates) and ST132 (14/133). Twenty-six OXA-51 allelic variants were detected, nine of which were novel. The PFGE types were generally susceptible (88/101) to all the tested antimicrobials; 3/101 were carbapenem-resistant due to the presence of the genetic structure IS*Aba2*-*bla*_OXA-58-like_-IS*Aba3*, and 2/101 were multidrug-resistant. It can be concluded that the examined Spo Acb complex population was mainly composed of *A. baumannii*. Many different clones were detected (with ST2 clearly dominant), all largely susceptible to antimicrobials; multidrug resistance was rare. In contrast, a previously examined EE Acb population was composed of just four expanding, multidrug-resistant *A. baumannii* clones -ST2, ST3, ST15, and ST80-.

## Introduction

The Acb complex has traditionally been regarded as formed by four *Acinetobacter* species: *Acinetobacter calcoaceticus*, *Acinetobacter baumannii*, *Acinetobacter nosocomialis*, and *Acinetobacter pittii* (Gerner-Smidt et al., 1991; Nemec et al., 2011). Other genomic species were later proposed for inclusion, including *Acinetobacter seifertii* [formerly *Acinetobacter* genomic species “close to 13TU” (Gerner-Smidt and Tjiernberg, 1993; Nemec et al., 2015)], *A. lactucae* (formerly *Acinetobacter* NB14 and synonym of *A. dijkshoorniae* (Espinal et al., 2015; Cosgaya et al., 2016; Rooney et al., 2016; Dunlap and Rooney, 2018), and the *Acinetobacter* genomic species “between 1 and 3.” All the species of the complex are genetically closely related and phenotypically indistinguishable; molecular methods are required for accurate identifications (Gerner-Smidt et al., 1991).

*Acinetobacter baumannii* is the species most frequently involved in nosocomial and community-acquired infections, followed by *A. pittii* and *A. nosocomialis* (Peleg et al., 2008). *A. calcoaceticus* has chiefly been isolated from environmental sources, such as soil and water, and its participation in clinical infections remains unclear. *A. seifertii*, *A. lactucae,* and *Acinetobacter* gen. species “between 1 and 3” have also been isolated from clinical specimens (Peleg et al., 2008; Nemec et al., 2015; Cosgaya et al., 2016).

Carbapenem-resistant *A. baumannii* has been involved in numerous nosocomial outbreaks around the world (Peleg et al., 2008; Higgins et al., 2010a; Antunes et al., 2014). This resistance is mainly determined by CHOs: OXA-23, OXA-40, and OXA-58) and less frequently by MBLs (Poirel and Nordmann, 2006; Peleg et al., 2008). At least eight *A. baumannii* CCs have been internationally disseminated, being CC1, CC2, and CC3 the most representative (Diancourt et al., 2010; Higgins et al., 2010a; Karah et al., 2012).

Sporadic and epidemic nosocomial strains of the Acb complex share the same ecological niche but show significant epidemiological differences. While the epidemic strains cause outbreaks (Peleg et al., 2008), the Spo strains are involved in the infection of individual patients, and for this reason have rarely been studied in any depth (Sonnevend et al., 2013; Selasi et al., 2016; Tomaschek et al., 2016).

The aim of the present work was to improve our knowledge of Spo nosocomial Acb complex populations, and thus better understand the epidemiology of the Spo strains in respect to the EE strains. The Spo population of the Acb complex at a single hospital over a 12-year period was characterized (species identification, clonal analysis, antimicrobial susceptibility, and carbapenem-resistance) and compared to its previously analyzed EE population (Villalón et al., 2015).

## Materials and Methods

### Background and Study Design

Three hundred and sixty-four Acb complex isolates were collected from a single tertiary care hospital during nosocomial outbreaks and interepidemic periods between 1999 and 2010. The clinical samples were taken as part of standard patient care. The bacterial isolates were involved in infectious diseases -except those obtained from screening of carriers and environmental sampling-, were previously identified by biochemical methods in the hospital and were sent to the Spanish National Centre for Microbiology (CNM) for their molecular characterization. All isolates were analyzed by PFGE, and 15 EE PFGE types (EE1 to EE15, 231 isolates) (Villalón et al., 2015) and 101 Spo PFGE types (Spo1 to Spo101, 133 isolates) obtained. Later, one representative isolate from each PFGE type was analyzed.

In a previous work (Villalón et al., 2015), we analyzed the 15 EE PFGE types. Now, this study shows three novelties in relation to that former work. First, we identified the species of the whole Acb complex population –i.e., Spo and EE PFGE types- using different molecular methods. Second, we undertook the molecular analysis of the 101 Spo PFGE types. Third, we compared the results from the Spo population with the preceding results (Villalón et al., 2015) from the EE population.

Some key molecular terms used within this study are next defined (Field et al., 2014): PFGE type or strain: a group of pathogens that share the same PFGE pattern. All distinguishable PFGE patterns were interpreted as different PFGE types or strains. Hereafter, the term “PFGE type” will be used. EE PFGE type: PFGE type that had ≥5 isolates from the same hospital ward at the same time, or PFGE type that although had <5 isolates showed ≥85% genetic similarity with any former PFGE type (Villalón et al., 2015). Spo PFGE type: PFGE type that did not fit into the EE definition. Isolate: each pure bacterial culture that was sent to the CNM for its analysis. This study included only one isolate per patient and infectious episode. Clone or ST: a group of PFGE types that shared the same MLST allelic profile. CC: a cluster of STs that showed one allele difference maximum (Diancourt et al., 2010).

Categorical variables were compared using the Chi-squared test with Mantel-Haenszel correction. Significance was set at *p* ≤ 0.05. All statistical analyses were performed using Epi Info software^1^.

### Molecular Identification of *Acinetobacter* Species: *gyr*B, *rpo*B, and *bla*_OXA-51-like_ Genes

PCR-amplification of *gyr*B polymorphisms was used to identify *A. baumannii* (294 and 490 bp fragments), *A. calcoaceticus* (428 bp), *A. nosocomialis* (294 bp), and *A. pittii* (194 bp); an additional 1194 bp fragment could also be obtained for *A. calcoaceticus*, *A. nosocomialis*, and *A. pittii* isolates (Higgins et al., 2007, 2010b). Sequencing of a *gyr*B 906 bp fragment was performed as described by the Clinical and Laboratory Standards Institute (CLSI) (Clinical and Laboratory Standards Institute, 2008). In addition, two hypervariable *rpo*B-fragments here named *rpo*B1 (zone 1, 351 bp) and *rpo*B2 (zone 2, 453 bp) (La Scola et al., 2006) were sequenced. All sequences were compared with type and reference strains in the GenBank database ^2^. Appropriate similarity cut-off for reliable species identification was used (94.0% for *gyr*B, *rpo*B1, and *rpo*B2) (Adékambi et al., 2008). Supplementary Table S1 shows the type and reference strains used. The *bla*_OXA-51-like_ gene was screened for confirmation of *A. baumannii* PFGE types (Woodford et al., 2006).

### Multilocus Sequence Typing

Multilocus sequence typing was performed following the Institute Pasteur protocol ^3^, although the primers used for *rpo*B were those described in Villalón et al. (2011). The partial *cpn60*, *fus*A, *glt*A, *pyr*G, *rec*A, *rpl*B, and *rpo*B genes were amplified using the conditions described by Bartual et al. (2005). Each allelic profile was assigned an ST.

### Antimicrobial Susceptibility and Carbapenemase Genes

Susceptibility testing for imipenem, meropenem, amikacin, gentamicin, tobramycin, ciprofloxacin, levofloxacin, trimethoprim-sulphamethoxazole, and colistin was undertaken using the MicroScan NM37 microdilution method (Dade Behring, West Sacramento, CA, United States). E-test strips (bioMérieux, Marcy-l’Etoile, France) were used to confirm imipenem resistance. The control strain used was *Pseudomonas aeruginosa* ATCC 27853. Results were interpreted using The European Committee on Antimicrobial Susceptibility Testing for *Acinetobacter* spp. (The European Committee on Antimicrobial Susceptibility Testing, 2017).

The complete *bla*_OXA-51-like_ gene (825 bp) was sequenced for all *A. baumannii* PFGE types; primers 51A (5′-ATGAACATTAAAGCACTCTTAC-3′) and 51D (5′-CTATAAAATACCTAATTGTTCT-3′) (Vahaboglu et al., 2006) amplified the gene; and primers 51A, OXA-51-likeFr&c (5′-CAAGGCGGATCAAAGCATTA-3′) (Turton et al., 2006) and OXA-51-OUT2 (5′-CCAAGATGAAGTGCAATCCA-3′) were used for sequencing (Evans, 2009).

The acquired CHO genes (*bla*_OXA-23-like_, *bla*_OXA-40-like_, and *bla*_OXA-58-like_), their most commonly associated insertion sequences (IS*Aba1*, IS*Aba2*, IS*Aba3*, IS*Aba4*, and IS*18*), and the MBL genes *bla*_IMP_, *bla*_V IM_, *bla*_SIM-1_, *bla*_GIM-1_, *bla*_SPM-1_, and *bla*_NDM-1_ were examined for imipenem-resistant PFGE types. The primers and PCR conditions used are described elsewhere (Villalón et al., 2013).

### Phylogenetic and Clonal Analysis

Sequences were assembled using Lasergene SeqMan II software (DNA Star, Inc., Madison, WI, United States). The phylogenetic clustering of *gyr*B, and *rpo*B1-*rpo*B2 concatenated partial sequences was constructed using the Neighbor-joining method, employing the Jukes-Cantor algorithm. Bootstrap values were obtained from 1000 resamplings. MLST clustering was represented using the MST method. Phylogenetic and MLST clustering was performed using BioNumerics software (version 5.1) (Applied Maths, Sint-Martens-Latem, Belgium).

### Ethics Statement

This study focused on bacteria and no identifiable human data were used, therefore ethical approval was exempted.

## Results

### Distribution of Sporadic *A. calcoaceticus-A. baumannii* Complex Isolates

One hundred and one PFGE types were detected among the 133 Spo isolates collected from the respiratory tract (51.1% of isolates), wounds (16.5%), urine (8.3%), samples for screening of *A. baumanni* carriers (8.3%), environmental samples (7.5%), blood (5.3%), biliary fluid (0.8%), and ‘not informed’ samples (2.3%). These isolates came from the pneumology ward on the third floor (42.9%), wards on the fourth floor (16.5%), the intensive care unit (10.5%), wards on the fifth floor (10.5%), the out-patient clinic consultation room (10.5%), the emergency room (5.3%), wards on the first floor (1.5%), and “not informed” locations (2.3%). Supplementary Table S2 shows the epidemiological data collected for all 101 Spo PFGE types.

### *gyr*B, *rpo*B, and *bla*_OXA-51-like_ Target Genes for Molecular Identification of *Acinetobacter* Species

Species (number of PFGE types) were identified as: *A. baumannii* (67), *A. pittii* (22), *A. lactucae* (6), *A. calcoaceticus* (2), *A. courvalinii* (1), *A. haemolyticus* (1), *A. proteolyticus* (1), and *A. schindleri* (1) (Supplementary Tables S1, S2 shows the results obtained by all three identification methods).

*gyr*B-PCR analysis (Higgins et al., 2007, 2010b) identified all *A. baumannii* (*n* = 67) and *A. pittii* (*n* = 22) PFGE types. *A. lactucae* PFGE types (*n* = 6) were misidentified: all returned an 1194 bp fragment and an additional 490 bp fragment was amplified for Spo100. With respect to *A. calcoaceticus* (*n* = 2), only Spo76 was misidentified since two 194 and 428 bp fragments were obtained. No DNA fragments were amplified for *Acinetobacter* species not included in the Acb complex.

*gyr*B sequencing returned 54 different sequences, 49 of which were novel. Comparisons with GenBank-held reference/type sequences revealed all PFGE types were reliably identified.

The sequencing of *rpo*B1 and *rpo*B2 returned 29 and 34 sequences, respectively, 13 and 16 of which were novel. All PFGE types were correctly identified, except for Spo56; which *rpo*B2 sequence shared only 92.8% genetic similarity with the *A. pittii* reference strain CIP 70.15 (Adékambi et al., 2008; Supplementary Table S1).

**FIGURE 1:**
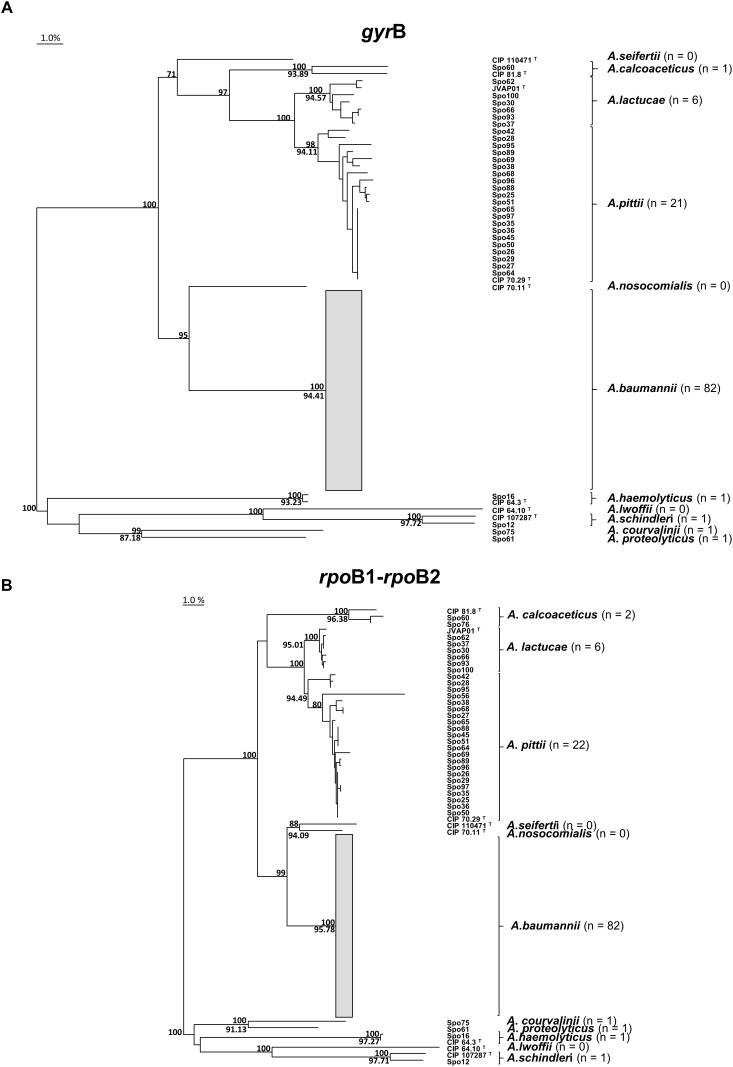
Clustering of *Acinetobacter* species. Phylogenetic trees obtained from the alignment of **(A)** partial *gyr*B gene sequences and **(B)** *rpo*B1-*rpo*B2 concatenated partial sequences. The trees were inferred by the Neighbor-joining method. Non-*Acinetobacter calcoaceticus-A. baumannii* complex PFGE types formed the outgroup to root the tree. Branches were supported by 1000 resamplings. Bootstrap values of ≥70% are indicated above branches. Minimum intraspecies similarity values (%) are shown below branches. The *A. baumannii* branch includes sporadic (Spo) and endemoepidemic (EE) PFGE types and is collapsed owing to its complexity. The bar indicates 1% genetic divergence. The total number of PFGE types (*n*) identified for each *Acinetobacter* species was calculated excluding the type (T) strains included in the analysis.

The novel *gyr*B, *rpo*B1, and *rpo*B2 sequences were each assigned a GenBank accession number (Supplementary Table S1). Figure 1 shows the phylogenetic trees of *gyr*B and the concatenated *rpo*B1-*rpo*B2 sequences, clustering all PFGE types into well-defined *Acinetobacter* species.

The *bla*_OXA-51-like_ gene was detected for *A. baumannii* (*n* = 67), but not for the non-*A. baumannii* PFGE types (*n* = 34).

### MLST Clonal Structure of the Sporadic Population

Sixty-three STs were detected for the 101 PFGE types, 38 of which were novel: ST687-ST697, ST832, ST833, ST849-ST872, and ST956. Seven PFGE types could not be typed due to a lack of PCR-amplification or to the presence of ambiguous nucleotides at some loci; these included one *A. baumannii* (Spo23), two *A. pittii* (Spo56 and Spo65), and the four non-Acb complex species identified (Supplementary Table S2). Sixty-six novel alleles were identified [with 56 (84.8%) among the non-A. *baumannii* PFGE types]; 15 for *cpn60* (*cpn60*-127 – *cpn60*-140 and *cpn60*-142), 10 for *fus*A (*fus*A-126 – *fus*A-135), nine for *glt*A (*glt*A-125 – *glt*A-133), five for *pyr*G (*pyr*G-68 – *pyr*G-72), 10 for *rec*A (*rec*A-138 – *rec*A-147), seven for *rpl*B (*rpl*B-70 – *rpl*B-76), and nine for *rpo*B (*rpo*B-121 – *rpo*B-129).

**FIGURE 2:**
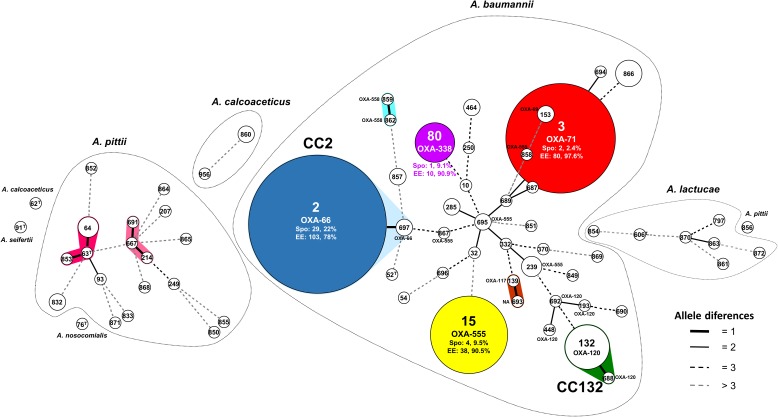
Minimum spanning tree showing the multilocus sequence typing (MLST) clonal structure of the Acb complex population. Circles represent the different STs; the size is proportional to the number of isolates. The connecting lines indicate the distance between STs, coded according to the number of locus variants. Dotted lines group the STs into *Acinetobacter* species. The clonal complexes are shadowed and cluster STs with a maximum of one different locus. Spo STs are represented in white. Colored STs include EE and Spo isolates (numbers and percentages indicated). The most representative OXA-51 alleles are shown for the *A. baumannii* group. CC, clonal complex; ST, sequence type; NA, not assigned; T, type strain; Spo, sporadic; EE, endemoepidemic.

The most common STs identified were ST2 (21.8% of isolates) and ST132 (10.5%). The remainder were represented by under 4% of isolates. Figure 2 shows the MST displaying the MLST clonal structure of the Acb complex population.

### Susceptibility to Antimicrobial Agents in Sporadic Population

Most (84.2%) of the Spo PFGE types were susceptible to all the tested antimicrobial agents. The total resistance rates were 3.0% for imipenem and meropenem, 7.0% for amikacin, 4.0% for gentamicin, 5.0% for tobramycin, 3.0% for ciprofloxacin, 4.0% for levofloxacin, 2.0% for trimethoprim-sulphamethoxazole, and 0% for colistin. Multidrug-resistance (MDR) (Magiorakos et al., 2012) was detected only for Spo1/ST2 and Spo2/ST2.

Exclusively Spo4/ST2, Spo32/ST10, and Spo94/ST15 showed carbapenem-resistance, but all three were susceptible to the remaining antimicrobial agents tested. Screening for acquired carbapenemases detected the genetic structure IS*Aba2*-*bla*_OXA-58-like_-IS*Aba3*. Sequencing of the complete *bla*_OXA-51-like_ gene revealed 26 allelic variants (GenBank accession numbers: KY126220 – KY126241), nine of which are here described for the first time (OXA-554 to OXA-562). Supplementary Table S2 describes the OXA-51 alleles detected, and Figure 2 shows the clonal distribution of the most common OXA-51 alleles.

### The *A. calcoaceticus-A. baumannii* Complex Population: Sporadic vs. Endemoepidemic

The Spo isolates were most commonly detected in respiratory samples (*p* < 0.001), and the EE isolates in the environmental (*p* < 0.001) and ICU-location (*p* = 0.03) samples. In addition, all the non-*A. baumannii* PFGE types were sporadic. ST2 was the most common in both populations, but the distribution of the other STs differed substantially. The Spo PFGE types returned a large number of STs, with 47 of the total 63 represented by one isolate only. In contrast, the EE PFGE types showed few STs (ST2, ST3, ST15, and ST80) that grouped a larger number of isolates (103, 80, 38, and 10, respectively) (Figure 2). Only two Spo PFGE types possessed MDR, while all the EE PFGE types showed either MDR (23 out of 231 isolates) or extensively drug-resistance (208 isolates) (Magiorakos et al., 2012; Villalón et al., 2015).

## Discussion

Nosocomial *A. baumannii* outbreaks are a major problem worldwide (Maragakis and Perl, 2008; Higgins et al., 2010a). Other *A. calcoaceticus-A. baumannii* complex species have been less frequently involved in epidemics, and have thus been less studied (Schleicher et al., 2012). Less still is known about the Spo Acb complex strains (Sonnevend et al., 2013) since these are not considered a serious public health problem. In this context, the present analysis contributes significantly toward our knowledge of the epidemiology and dynamics of the Acb complex as a whole.

Most Spo isolates (82%) were involved in nosocomial infections. The respiratory tract was confirmed as the most common site of Acb complex nosocomial infections (Peleg et al., 2008), although in the present work 51% of Spo and 32% of EE isolates were obtained from respiratory samples (*p* < 0.001). The strict surveillance implemented on the pneumology ward - affected by one outbreak and by the endemic persistence of several clones (Villalón et al., 2015) – might explain the large number of Spo isolates detected in samples of this origin. In addition, the EE isolates were more commonly retrieved from environmental samples (*p* < 0.001), probably because environmental sampling is routinely performed during outbreaks (Peleg et al., 2008). Finally, EE strains were more commonly retrieved from the ICU (the ward most affected by *A. baumannii* epidemics) than were Spo strains (*p* = 0.03).

*Acinetobacter baumannii* was the major species detected among the Spo PFGE types, followed by *A. pittii*, *A. lactucae*, and *A. calcoaceticus*. However, other important species like *A. nosocomialis*, *A. seifertii*, and *Acinetobacter* gen. species “between 1 and 3” were absent. Further, *A. baumanni* was the only species detected among the EE PFGE types (Villalón et al., 2015). These results agree with *A. baumannii* to be the species of the Acb complex mainly involved in either outbreaks or Spo infections (Peleg et al., 2008).

The *gyr*B multiplex PCR analysis was designed to differentiate *A. baumannii*, *A. calcoaceticus*, *A. nosocomialis*, and *A. pittii* (Higgins et al., 2007, 2010b) only. *A. baumannii* and *A. pittii* PFGE types were correctly identified, but one out of two *A. calcoaceticus* (Spo76), and all of the *A. lactucae* PFGE types, were misidentified. The results were expected except for Spo76.

Matching of the *gyr*B sequences with those in databases identified all PFGE types correctly. The identification of *A. lactucae* was more troublesome than the other species due to the lack of *gyr*B reference entries. We also detected wrong identifications in GenBank database, like actual *A.pittii* misidentified as *A. calcoaceticus*, and actual *A. lactucae* misidentified as *A. pittii*; it should be considered in this kind of analyses. However, all but one PFGE type were reliably identified by *rpoB*1 and *rpoB*2 sequencing. *gyr*B sequencing has slight disadvantages compared to *rpo*B1- and *rpo*B2-sequencing: the lack of database for *A. lactucae* and the extra work required to sequence the larger genetic fragment. Both *rpo*B1 and *rpo*B2 analyses seem able to accurately identify *Acinetobacter* species, but *rpo*B1 fragment is shorter, making its use more practical (La Scola et al., 2006). Finally, phylogenetic analysis of the *gyr*B and *rpo*B1-*rpo*B2 concatenated sequences showed monophyletic clustering for each species of the Acb complex (Figure 1), which reflects the excellent discrimination power for both methods.

Given the present results, the PCR-amplification of *gyr*B is here proposed as a first step in the molecular identification of Acb complex members, because it efficiently identifies at least two of the most frequent species -*A. baumannii, and A. pittii*-. If the results obtained are not conclusive, the sequencing of *rpo*B1, *rpo*B2, and the partial *gyr*B gene should be performed in the order described. If sequence-matching with information in databases fails to provide reliable results, a final phylogenetic analysis should be performed to make definitive species identifications.

In the present study, we have purposely undertaken the identification at the species level using molecular methods only, and we have shown the complexity of such identification due to the close genetic relatedness of members of the Acb complex in particular when trying to differentiate between *A. pittii* and *A. lactucae*. However, *A. nosocomialis* and *A. seifertii* were not isolated in this setting, and it was not possible to check if similar difficulty also affected differentiation of these two closely related species. We are aware that the recent implementation of MALDI-TOF/MS has recently been shown to solve some of these issues but such technology is not yet easily available to all routine clinical laboratories.

The MLST clonal structure of the Acb complex grouped the four identified species into different clusters (Figure 2). The Acb complex Spo population was highly diverse, with a large number of STs mostly represented by single isolates. Other authors have also described this variability in Spo clones (Karah et al., 2012; Sonnevend et al., 2013; Tomaschek et al., 2016). The *A. baumannii* Spo population grouped into 37 STs, with ST2 and ST132 the most prevalent. The international clonal complex CC2 was represented by ST2 and ST697, and carried the OXA-66 allele (*bla*_OXA-51-like_ gene). CC132 was mainly represented by ST132 and carried the OXA-120 allele. The number of isolates and its distribution from 2000 to 2009 suggest that ST132 should be monitored for the emergence of greater resistance. Other international CCs detected in Spo isolates were represented by ST15 and ST10. Some OXA-51 alleles were detected that were not always linked to a specific CC (Pournaras et al., 2014): OXA-555 was carried by ST15 and by other non-related STs, and OXA-120 was detected in three STs not included in CC132 (Figure 2). The *A. pittii* population showed only two small CCs, one with ST64 as the most common ST. *A. lactucae* and *A. calcoaceticus* grouped into no CCs. The *A. baumannii* EE PFGE types, in contrast, grouped into four STs (ST2, ST3, ST15, and ST80: Figure 2), each represented by a large number of isolates (Villalón et al., 2015).

OXA-58, one of the main CHOs in *A. baumannii* in Spain (Villalón et al., 2013; Nowak et al., 2017), was responsible for the detected resistance to imipenem in Spo clones. MDR and carbapenem resistance was associated to the international ST2, ST10, and ST15 (Diancourt et al., 2010). Antimicrobial resistance and other virulence factors contribute to the epidemicity of the strains (Selasi et al., 2016). In this study, resistance and epidemicity are closely related and so, the presence of Spo resistant clones in the hospital setting could be a risk for future outbreaks (Fournier and Richet, 2006; Sonnevend et al., 2013).

In regards to the EE clones; ST2 belongs to the international CC2, the most frequent in many European countries, and notably in the Mediterranean area. The high presence of ST2 conditioned its majority in Spo isolates. ST3 is the main representative of the international CC3; its global distribution is lesser than international CC1 and CC2, but in Spain it has been more frequently isolated than the CC1. ST15 belongs to another international clonal lineage and has an intermediate distribution in Europe. ST80 was first described in Spain where was involved in some nosocomial outbreaks, but it has not been described as an international clonal lineage at the moment (Higgins et al., 2010a; Villalón et al., 2011; Karah et al., 2012; Castanheira et al., 2014; Tomaschek et al., 2016; Dahdouh et al., 2017; Nowak et al., 2017).

Resistance to carbapenems was common in EE clones and it was due to the presence of *bla*_OXA-40-like_, and *bla*_OXA-58-like_ genes. OXA-40 and OXA-58 were the most frequent CHOs detected in *A. baumannii* in Spain during the period 1999–2010, which is in excellent agreement with the epidemiology and the main mechanisms of carbapenem resistance detected in our isolates, also recovered within the same time frame. Nevertheless, in 2013 the first isolate carrying *bla*_OXA-23-like_ was detected in our country and its presence has gradually increased ever since (Espinal et al., 2013; Mosqueda et al., 2013; Dahdouh et al., 2017). Currently, the replacement of *bla*_OXA-40-like_ by *bla*_OXA-23-like_ could be happening as it was previously described in Portugal (Grosso et al., 2011). Unfortunately, it was not possible to include in our study isolates recovered after 2011 and, therefore, we could not evaluate the impact of recent changes in the epidemiology of *Acinetobacter* in Spain on the dynamics of EE and Spo clones in this particular setting.

In conclusion, the Spo Acb complex population examined was diverse, though it was mainly composed of *A. baumannii*. The clonal structure reflected many different clones, although none but CC2 was clearly dominant. Most clones were susceptible to antimicrobial agents; MDR was very rare. On the other hand, the EE Acb population was more homogeneous, made up of a few expanding *A. baumannii* CCs showing MDR.

## Author Contributions

PV, JS-N, and SV conceived the study. PV, MO, GC, MM-P, NG, and SV analyzed and investigated the data. JS-N and SV supervised the study. PV and SV wrote the manuscript.

## Conflict of Interest Statement

The authors declare that the research was conducted in the absence of any commercial or financial relationships that could be construed as a potential conflict of interest.

## Footnotes

1 https://www.cdc.gov/epiinfo

2 www.ncbi.nlm.nih.gov/

3 www.pubmlst.org

## Abbreviations

Acb: *Acinetobacter calcoaceticus – Acinetobacter baumannii*
CC: clonal complex
CHO: carbapenem-hydrolysing oxacillinase
EE: endemoepidemic
MBL: metallo-beta-lactamase
MDR: multidrug resistance
MLST: multilocus sequence typing
MST: minimum spanning tree
PFGE: pulsed-field gel electrophoresis
Spo: sporadic
ST: sequence type
