# Supplementary material for: Dynamics of a Sporadic Nosocomial Acinetobacter calcoaceticus – Acinetobacter baumannii Complex Population
Source: Front Microbiol. 2019 Mar 22;10:593. doi: 10.3389/fmicb.2019.00593 (PMC6440288; doi:10.3389/fmicb.2019.00593)
Supplement: Supplementary file 1 [file Data_Sheet_1.PDF]

Supplementary Table 1. Sporadic (Spo) and endemoepidemic (EE) *Acinetobacter* spp. PFGE types: identification of species by *gyr*B-PCR (1, 2), *gyr*B-sequencing (3) and *rpo*B-sequencing (4)

PFGE type	*gyr*B PCR Identification	*gyr*B sequencing				*rpo*B1 sequencing				*rpo*B2 sequencing			
		Identification[a]	GenBank acc. no.[b]	Type/ref. strain[c]	% ident[a]	Identification	GenBank acc. no.[b]	Type/ref. strain[c]	% ident[a]	Identification	GenBank acc. no.[b]	Type/ref. strain[c]	% ident[a]
EE1	*A. baumannii*	*A. baumannii*		ACICU	99.8	*A. baumannii*	KY126157	ACICU	100	*A. baumannii*	KY126186	ACICU	100
EE2	*A. baumannii*	*A. baumannii*		ACICU	100	*A. baumannii*	KY126157	ACICU	100	*A. baumannii*	KY126186	ACICU	100
EE3	*A. baumannii*	*A. baumannii*		ACICU	100	*A. baumannii*	KY126157	ACICU	100	*A. baumannii*	KY126186	ACICU	100
EE4	*A. baumannii*	*A. baumannii*		ACICU	100	*A. baumannii*	KY126157	ACICU	100	*A. baumannii*	KY126186	ACICU	100
EE5	*A. baumannii*	*A. baumannii*		ACICU	100	*A. baumannii*	KY126157	ACICU	100	*A. baumannii*	KY126186	ACICU	100
EE6	*A. baumannii*	*A. baumannii*		ACICU	100	*A. baumannii*	KY126157	ACICU	100	*A. baumannii*	KY126186	ACICU	100
EE7	*A. baumannii*	*A. baumannii*		ACICU	100	*A. baumannii*	KY126157	ACICU	100	*A. baumannii*	KY126186	ACICU	100
EE8	*A. baumannii*	*A. baumannii*		ACICU	100	*A. baumannii*	KY126157	ACICU	100	*A. baumannii*	KY126186	ACICU	100
EE9	*A. baumannii*	*A. baumannii*	**KY126135**	ACICU	99.9	*A. baumannii*	KY126157	ACICU	100	*A. baumannii*	KY126203	CIP 70.34[T]	99.8
EE10	*A. baumannii*	*A. baumannii*	**KY126146**	ATCC 17978	99.2	*A. baumannii*	KY126157	ACICU	100	*A. baumannii*	KY126186	ACICU	100
EE11	*A. baumannii*	*A. baumannii*	**KY126122**	CIP 70.22	99.6	*A. baumannii*	KY126157	ACICU	100	*A. baumannii*	KY126197	NIPH1734	100
EE12	*A. baumannii*	*A. baumannii*	**KY126111**	CIP 70.34 [T]	99.3	*A. baumannii*	KY126160	LUH 5875	100	*A. baumannii*	KY126189	CIP 70.34[T]	100
EE13	*A. baumannii*	*A. baumannii*	**KY126111**	CIP 70.34 [T]	99.3	*A. baumannii*	KY126160	LUH 5875	100	*A. baumannii*	KY126189	CIP 70.34[T]	100
EE14	*A. baumannii*	*A. baumannii*	**KY126111**	CIP 70.34 [T]	99.3	*A. baumannii*	KY126160	LUH 5875	100	*A. baumannii*	KY126189	CIP 70.34[T]	100
EE15	*A. baumannii*	*A. baumannii*	**KY126111**	CIP 70.34 [T]	99.3	*A. baumannii*	KY126160	LUH 5875	100	*A. baumannii*	KY126189	CIP 70.34[T]	100
Spo1	*A. baumannii*	*A. baumannii*		ACICU	100	*A. baumannii*	KY126157	ACICU	100	*A. baumannii*	KY126186	ACICU	100
Spo2	*A. baumannii*	*A. baumannii*		ACICU	100	*A. baumannii*	KY126157	ACICU	100	*A. baumannii*	KY126186	ACICU	100
Spo3	*A. baumannii*	*A. baumannii*		ACICU	100	*A. baumannii*	KY126157	ACICU	100	*A. baumannii*	KY126186	ACICU	100
Spo4	*A. baumannii*	*A. baumannii*		ACICU	100	*A. baumannii*	KY126157	ACICU	100	*A. baumannii*	KY126186	ACICU	100
Spo5	*A. baumannii*	*A. baumannii*		ACICU	100	*A. baumannii*	KY126157	ACICU	100	*A. baumannii*	KY126186	ACICU	100
Spo6	*A. baumannii*	*A. baumannii*	**KY126107**	CIP 70.10	99.4	*A. baumannii*	KY126158	CIP 70.10	100	*A. baumannii*	KY126187	CIP 70.10	100
Spo7	*A. baumannii*	*A. baumannii*		ACICU	100	*A. baumannii*	KY126157	ACICU	100	*A. baumannii*	KY126186	ACICU	100
Spo8	*A. baumannii*	*A. baumannii*	**KY126108**	ATCC 17978	99.9	*A. baumannii*	KY126157	ACICU	100	*A. baumannii*	KY126188	SDF	100
Spo9	*A. baumannii*	*A. baumannii*		ACICU	100	*A. baumannii*	KY126157	ACICU	100	*A. baumannii*	KY126186	ACICU	100
Spo10	*A. baumannii*	*A. baumannii*	**KY126109**	ACICU	99.1	*A. baumannii*	KY126159	ATCC17904	100	*A. baumannii*	KY126186	ACICU	100
Spo11	*A. baumannii*	*A. baumannii*	**KY126110**	ACICU	99.9	*A. baumannii*	KY126157	ACICU	100	*A. baumannii*	KY126186	ACICU	100
Spo12	Negative	*A. schindleri*		ACE	99.2	*A. schindleri*	**KY126161**	CIP107287[T]	94.3	*A. schindleri*	**KY126190**	CIP107287[T]	98.9
Spo13	*A. baumannii*	*A. baumannii*		ACICU	100	*A. baumannii*	KY126157	ACICU	100	*A. baumannii*	KY126186	ACICU	100
Spo14	*A. baumannii*	*A. baumannii*	**KY126112**	SDF	99.9	*A. baumannii*	KY126162	LUH 4708	100	*A. baumannii*	KY126191	CIP 70.34 [T]	99.8
Spo15	*A. baumannii*	*A. baumannii*	**KY126107**	CIP 70.10	99.4	*A. baumannii*	KY126159	ATCC17904	100	*A. baumannii*	KY126186	ACICU	100
Spo16	Negative	*A.haemolyticus*	**KY126113**	CIP 64.3 [T]	99.6	*A.haemolyticus*	KY126163	CIP 64.3 [T]	100	*A.haemolyticus*	KY126192	CIP 64.3 [T]	99.7
Spo17	*A. baumannii*	*A. baumannii*	**KY126114**	CIP 70.10	99.9	*A. baumannii*	KY126164	NIPH 601	100	*A. baumannii*	KY126204	ATCC17904	99.8
Spo18	*A. baumannii*	*A. baumannii*	**KY126112**	SDF	99.9	*A. baumannii*	KY126158	CIP 70.10	100	*A. baumannii*	KY126187	CIP 70.10	100
Spo19	*A. baumannii*	*A. baumannii*		ACICU	100	*A. baumannii*	KY126157	ACICU	100	*A. baumannii*	KY126186	ACICU	100
Spo20	*A. baumannii*	*A. baumannii*	**KY126107**	CIP 70.10	99.4	*A. baumannii*	KY126158	CIP 70.10	100	*A. baumannii*	KY126187	CIP 70.10	100
Spo21	*A. baumannii*	*A. baumannii*	**KY126107**	CIP 70.10	99.4	*A. baumannii*	KY126158	CIP 70.10	100	*A. baumannii*	KY126187	CIP 70.10	100

PFGE type	*gyr*B PCR Identification	*gyr*B sequencing				*rpo*B1 sequencing				*rpo*B2 sequencing			
		Identification[a]	GenBank acc. no.[b]	Type/ref. strain[c]	% ident[a]	Identification	GenBank acc. no.[b]	Type/ref. strain[c]	% ident[a]	Identification	GenBank acc. no.[b]	Type/ref. strain[c]	% ident[a]
Spo22	*A. baumannii*	*A. baumannii*	**KY126115**	ATCC 17978	99.4	*A. baumannii*	KY126158	CIP 70.10	100	*A. baumannii*	KY126187	CIP 70.10	100
Spo23	*A. baumannii*	*A. baumannii*	**KY126116**	SDF	99.7	*A. baumannii*	KY126158	CIP 70.10	100	*A. baumannii*	KY126189	CIP 70.34 ^{T}	100
Spo24	*A. baumannii*	*A. baumannii*	**KY126117**	CIP 70.34 ^{T}	99.6	*A. baumannii*	KY126164	NIPH 601	100	*A. baumannii*	KY126186	ACICU	100
Spo25	*A. pittii*	*A. pittii*	**KY126118**	CIP 70.29 ^{T}	99.2	*A. pittii*	KY126165	CIP 70.29 ^{T}	100	*A. pittii*	KY126193	LMG 10562	100
Spo26	*A. pittii*	*A. pittii*		CIP 70.29 ^{T}	100	*A. pittii*	KY126165	CIP 70.29 ^{T}	100	*A. pittii*	KY126193	LMG 10562	100
Spo27	*A. pittii*	*A. pittii*		CIP 70.29 ^{T}	100	*A. pittii*	**KY126166**	CIP 70.15	99.4	*A. pittii*	**KY126194**	LMG 10560	99.6
Spo28	*A. pittii*	*A. pittii*	**KY126119**	PHEA-2	97.1	*A. pittii*	**KY126167**	CIP 70.29 ^{T}	99.1	*A. pittii*	**KY126195**	ANC 4626	99.6
Spo29	*A. pittii*	*A. pittii*		CIP 70.29 ^{T}	100	*A. pittii*	KY126165	CIP 70.29 ^{T}	100	*A. pittii*	KY126193	LMG 10562	100
Spo30	1194 pb	*A.lactucae*	**KY126120**	JVPA01 ^{T}	98.3	*A.lactucae*	KY126168	LUH 7351	100	*A.lactucae*	KY126196	LUH13626	100
Spo31	*A. baumannii*	*A. baumannii*	**KY126121**	SDF	99.8	*A. baumannii*	KY126162	LUH 4708	100	*A. baumannii*	KY126191	CIP 70.34 ^{T}	99.8
Spo32	*A. baumannii*	*A. baumannii*		CIP 70.10	100	*A. baumannii*	KY126169	NIPH 335	100	*A. baumannii*	KY126188	SDF	100
Spo33	*A. baumannii*	*A. baumannii*	**KY126123**	ATCC 17978	99.3	*A. baumannii*	KY126158	CIP 70.10	100	*A. baumannii*	KY126187	CIP 70.10	100
Spo34	*A. baumannii*	*A. baumannii*	**KY126124**	ATCC 17978	99.1	*A. baumannii*	KY126170	ATCC17978	100	*A. baumannii*	KY126186	ACICU	100
Spo35	*A. pittii*	*A. pittii*		CIP 70.29 ^{T}	100	*A. pittii*	KY126165	CIP 70.29 ^{T}	100	*A. pittii*	**KY126198**	LMG 10562	99.8
Spo36	*A. pittii*	*A. pittii*		CIP 70.29 ^{T}	100	*A. pittii*	KY126165	CIP 70.29 ^{T}	100	*A. pittii*	KY126193	LMG 10562	100
Spo37	1194 pb	*A.lactucae*	**KY126125**	ANC 4052	98.0	*A.lactucae*	KY126168	LUH 7351	100	*A.lactucae*	KY126196	LUH13626	100
Spo38	*A. pittii*	*A. pittii*	**KY126126**	PHEA-2	99.6	*A. pittii*	KY126171	PHEA-2	100	*A. pittii*	**KY126199**	ANC 5638	99.6
Spo39	*A. baumannii*	*A. baumannii*		ACICU	100	*A. baumannii*	KY126157	ACICU	100	*A. baumannii*	KY126186	ACICU	100
Spo40	*A. baumannii*	*A. baumannii*		ACICU	100	*A. baumannii*	KY126157	ACICU	100	*A. baumannii*	KY126186	ACICU	100
Spo41	*A. baumannii*	*A. baumannii*	**KY126121**	SDF	99.8	*A. baumannii*	**KY126172**	ATCC17978	99.7	*A. baumannii*	KY126189	CIP 70.34 ^{T}	100
Spo42	*A. pittii*	*A. pittii*	**KY126127**	PHEA-2	97.4	*A. pittii*	**KY126173**	CIP 70.29 ^{T}	99.7	*A. pittii*	**KY126200**	ANC 4626	99.6
Spo43	*A. baumannii*	*A. baumannii*	**KY126107**	CIP 70.10	99.4	*A. baumannii*	KY126158	CIP 70.10	100	*A. baumannii*	KY126187	CIP 70.10	100
Spo44	*A. baumannii*	*A. baumannii*	**KY126128**	ATCC 17978	99.1	*A. baumannii*	KY126170	ATCC17978	100	*A. baumannii*	KY126189	CIP 70.34 ^{T}	100
Spo45	*A. pittii*	*A. pittii*		CIP 70.29 ^{T}	100	*A. pittii*	KY126174	CIP 70.15	100	*A. pittii*	KY126201	CIP 70.15	100
Spo46	*A. baumannii*	*A. baumannii*	**KY126129**	ATCC 17978	99.4	*A. baumannii*	KY126159	ATCC17904	100	*A. baumannii*	KY126186	ACICU	100
Spo47	*A. baumannii*	*A. baumannii*	**KY126130**	ATCC 17978	99.4	*A. baumannii*	KY126170	ATCC17978	100	*A. baumannii*	KY126189	CIP 70.34 ^{T}	100
Spo48	*A. baumannii*	*A. baumannii*	**KY126131**	CIP 70.10	99.7	*A. baumannii*	KY126158	CIP 70.10	100	*A. baumannii*	KY126187	CIP 70.10	100
Spo49	*A. baumannii*	*A. baumannii*		ACICU	100	*A. baumannii*	KY126157	ACICU	100	*A. baumannii*	KY126186	ACICU	100
Spo50	*A. pittii*	*A. pittii*		CIP 70.29 ^{T}	100	*A. pittii*	KY126165	CIP 70.29 ^{T}	100	*A. pittii*	KY126193	LMG 10562	100
Spo51	*A. pittii*	*A. pittii*	**KY126132**	CIP 70.29 ^{T}	99.4	*A. pittii*	KY126174	CIP 70.15	100	*A. pittii*	KY126201	CIP 70.15	100
Spo52	*A. baumannii*	*A. baumannii*		ACICU	100	*A. baumannii*	KY126157	ACICU	100	*A. baumannii*	KY126186	ACICU	100
Spo53	*A. baumannii*	*A. baumannii*	**KY126107**	CIP 70.10	99.4	*A. baumannii*	KY126158	CIP 70.10	100	*A. baumannii*	KY126187	CIP 70.10	100
Spo54	*A. baumannii*	*A. baumannii*	**KY126133**	CIP 70.34 ^{T}	99.7	*A. baumannii*	KY126160	LUH 5875	100	*A. baumannii*	KY126188	SDF	100
Spo55	*A. baumannii*	*A. baumannii*	**KY126107**	CIP 70.10	99.4	*A. baumannii*	KY126158	CIP 70.10	100	*A. baumannii*	KY126187	CIP 70.10	100
Spo56	*A. pittii*	NA[d]		-[d]	-[d]	*A. pittii*	KY126171	PHEA-2	100	*Acinetobacter* spp.	**KY126202**	CIP 70.15	92.8
Spo57	*A. baumannii*	*A. baumannii*	**KY126134**	ACICU	99.0	*A. baumannii*	**KY126175**	ACICU	99.7	*A. baumannii*	KY126189	CIP 70.34 ^{T}	100
Spo58	*A. baumannii*	*A. baumannii*	**KY126135**	ACICU	99.9	*A. baumannii*	KY126157	ACICU	100	*A. baumannii*	KY126203	CIP 70.34 ^{T}	99.8
Spo59	*A. baumannii*	*A. baumannii*	**KY126136**	CIP 70.34 ^{T}	99.4	*A. baumannii*	KY126164	NIPH 601	100	*A. baumannii*	KY126204	NIPH 67	100
Spo60	*A.calcoaceticus*	*A.calcoaceticus*	**KY126137**	CIP 81.08 ^{T}	94.6	*A.calcoaceticus*	**KY126176**	ANC 4221	99.7	*A.calcoaceticus*	**KY126205**	ANC 4221	99.6

PFGE type	*gyr*B PCR Identification	*gyr*B sequencing				*rpo*B1 sequencing				*rpo*B2 sequencing			
		Identification[a]	GenBank acc. no.[b]	Type/ref. strain[c]	% ident[a]	Identification	GenBank acc. no.[b]	Type/ref. strain[c]	% ident[a]	Identification	GenBank acc. no.[b]	Type/ref. strain[c]	% ident[a]
Spo61	Negative	*A.proteolyticus*	**KY126138**	NIPH 809 ^{T}	99.2	*A.proteolyticus*	KY126177	ANC 3839	100	*A.proteolyticus*	**KY126206**	ANC 3839	98.9
Spo62	1194 pb	*A.lactucae*	**KY126139**	JVPA01 ^{T}	99.6	*A.lactucae*	**KY126178**	LUH 7351	99.7	*A.lactucae*	KY126196	LUH13626	100
Spo63	*A. baumannii*	*A. baumannii*	**KY126134**	ACICU	99.0	*A. baumannii*	**KY126175**	ACICU	99.7	*A. baumannii*	KY126189	CIP 70.34 ^{T}	100
Spo64	*A. pittii*	*A. pittii*		CIP 70.29 ^{T}	100	*A. pittii*	KY126174	CIP 70.15	100	*A. pittii*	KY126201	CIP 70.15	100
Spo65	*A. pittii*	*A. pittii*		CIP 70.29 ^{T}	100	*A. pittii*	KY126165	CIP 70.29 ^{T}	100	*A. pittii*	**KY126207**	NIPH 2805	99.6
Spo66	1194 pb	*A.lactucae*	**KY126140**	ANC 4052	97.8	*A.lactucae*	**KY126179**	LUH 10243	99.7	*A.lactucae*	KY126196	LUH13626	100
Spo67	*A. baumannii*	*A. baumannii*		ACICU	100	*A. baumannii*	KY126157	ACICU	100	*A. baumannii*	KY126186	ACICU	100
Spo68	*A. pittii*	*A. pittii*	**KY126141**	CIP 70.29 ^{T}	98.8	*A. pittii*	**KY126166**	CIP 70.15	99.4	*A. pittii*	**KY126194**	LMG 10560	99.6
Spo69	*A. pittii*	*A. pittii*	**KY126142**	PHEA-2	98.9	*A. pittii*	KY126180	LMG 10559	100	*A. pittii*	KY126208	NIPH 2805	100
Spo70	*A. baumannii*	*A. baumannii*		ACICU	100	*A. baumannii*	KY126157	ACICU	100	*A. baumannii*	KY126186	ACICU	100
Spo71	*A. baumannii*	*A. baumannii*	**KY126122**	CIP 70.22	99.6	*A. baumannii*	KY126157	ACICU	100	*A. baumannii*	KY126197	NIPH 1734	100
Spo72	*A. baumannii*	*A. baumannii*	**KY126143**	ATCC 17978	99.3	*A. baumannii*	**KY126181**	ATCC17978	99.7	*A. baumannii*	**KY126209**	CIP 70.34 ^{T}	99.6
Spo73	*A. baumannii*	*A. baumannii*	**KY126144**	CIP 70.22	99.4	*A. baumannii*	**KY126182**	ACICU	99.4	*A. baumannii*	KY126210	CIP 70.34 ^{T}	99.8
Spo74	*A. baumannii*	*A. baumannii*		ACICU	100	*A. baumannii*	KY126157	ACICU	100	*A. baumannii*	KY126186	ACICU	100
Spo75	Negative	*A. courvalinii*	**KY126145**	ANC 4230	97.9	*A. courvalinii*	**KY126183**	NIPH 1850	99.7	*A. courvalinii*	**KY126211**	ANC 4930	99.8
Spo76	200 & 428 pb	NA[d]		-[d]	-[d]	*A.calcoaceticus*	**KY126184**	ANC 41.04	98.9	*A.calcoaceticus*	**KY126212**	ANC 3811	98.5
Spo77	*A. baumannii*	*A. baumannii*	**KY126146**	ATCC 17978	99.2	*A. baumannii*	KY126170	ATCC17978	100	*A. baumannii*	KY126189	CIP 70.34 ^{T}	100
Spo78	*A. baumannii*	*A. baumannii*		ACICU	100	*A. baumannii*	KY126157	ACICU	100	*A. baumannii*	KY126186	ACICU	100
Spo79	*A. baumannii*	*A. baumannii*	**KY126146**	ATCC 17978	99.2	*A. baumannii*	KY126157	ACICU	100	*A. baumannii*	KY126186	ACICU	100
Spo80	*A. baumannii*	*A. baumannii*	**KY126147**	CIP 70.22	99.6	*A. baumannii*	KY126170	ATCC17978	100	*A. baumannii*	KY126204	NIPH 67	100
Spo81	*A. baumannii*	*A. baumannii*	**KY126148**	CIP 70.34 ^{T}	99.3	*A. baumannii*	KY126169	NIPH 335	100	*A. baumannii*	KY126189	CIP 70.34 ^{T}	100
Spo82	*A. baumannii*	*A. baumannii*	**KY126149**	ACICU	99.8	*A. baumannii*	**KY126172**	ATCC17978	99.7	*A. baumannii*	KY126186	ACICU	100
Spo83	*A. baumannii*	*A. baumannii*	**KY126107**	CIP 70.10	99.4	*A. baumannii*	KY126158	CIP 70.10	100	*A. baumannii*	KY126187	CIP 70.10	100
Spo84	*A. baumannii*	*A. baumannii*	**KY126112**	SDF	99.9	*A. baumannii*	KY126158	CIP 70.10	100	*A. baumannii*	KY126187	CIP 70.10	100
Spo85	*A. baumannii*	*A. baumannii*	**KY126150**	ATCC 17978	99.6	*A. baumannii*	KY126170	ATCC17978	100	*A. baumannii*	**KY126213**	CIP 70.34 ^{T}	99.8
Spo86	*A. baumannii*	*A. baumannii*	**KY126111**	CIP 70.34 ^{T}	99.3	*A. baumannii*	KY126160	LUH 5875	100	*A. baumannii*	KY126189	CIP 70.34 ^{T}	100
Spo87	*A. baumannii*	*A. baumannii*		ACICU	100	*A. baumannii*	KY126157	ACICU	100	*A. baumannii*	KY126186	ACICU	100
Spo88	*A. pittii*	*A. pittii*		CIP 70.29 ^{T}	99.3	*A. pittii*	KY126174	CIP 70.15	100	*A. pittii*	KY126201	CIP 70.15	100
Spo89	*A. pittii*	*A. pittii*	**KY126151**	PHEA-2	99.6	*A. pittii*	KY126165	CIP 70.29 ^{T}	100	*A. pittii*	**KY126214**	LMG 10560	99.6
Spo90	*A. baumannii*	*A. baumannii*	**KY126147**	ATCC 17978	99.2	*A. baumannii*	KY126169	NIPH 335	100	*A. baumannii*	KY126189	CIP 70.34 ^{T}	100
Spo91	*A. baumannii*	*A. baumannii*	**KY126149**	ACICU	99.8	*A. baumannii*	**KY126172**	ATCC17978	99.7	*A. baumannii*	KY126186	ACICU	100
Spo92	*A. baumannii*	*A. baumannii*	**KY126152**	ATCC 17978	99.2	*A. baumannii*	**KY126172**	ATCC17978	99.7	*A. baumannii*	KY126186	ACICU	100
Spo93	1194 pb	*A.lactucae*	**KY126153**	ANC 4052	97.9	*A.lactucae*	KY126185	LUH 10243	100	*A.lactucae*	**KY126215**	LUH 7351	99.6
Spo94	*A. baumannii*	*A. baumannii*	**KY126122**	CIP 70.22	99.6	*A. baumannii*	KY126157	ACICU	100	*A. baumannii*	KY126197	NIPH 1734	100
Spo95	*A. pittii*	*A. pittii*	**KY126154**	PHEA-2	98.2	*A. pittii*	**KY126173**	CIP 70.29 ^{T}	99.7	*A. pittii*	**KY126195**	ANC 4626	99.6
Spo96	*A. pittii*	*A. pittii*	**KY126155**	CIP 70.29 ^{T}	99.1	*A. pittii*	KY126165	CIP 70.29 ^{T}	100	*A. pittii*	KY126217	LMG 10560	100
Spo97	*A. pittii*	*A. pittii*		CIP 70.29 ^{T}	100	*A. pittii*	KY126165	CIP 70.29 ^{T}	100	*A. pittii*	**KY126198**	LMG 10562	99.8
Spo98	*A. baumannii*	*A. baumannii*	**KY126111**	CIP 70.34 ^{T}	99.3	*A. baumannii*	KY126160	LUH 5875	100	*A. baumannii*	KY126189	CIP 70.34 ^{T}	100
Spo99	*A. baumannii*	*A. baumannii*	**KY126112**	SDF	99.9	*A. baumannii*	KY126162	LUH 4708	100	*A. baumannii*	KY126191	CIP 70.34 ^{T}	99.8

PFGE type	gyrB PCR Identification	*gyr*B sequencing				*rpo*B1 sequencing				*rpo*B2 sequencing			
		Identification[a]	GenBank acc. no.[b]	Type/ref. strain[c]	% ident[a]	Identification	GenBank acc. no.[b]	Type/ref. strain[c]	% ident[a]	Identification	GenBank acc. no.[b]	Type/ref. strain[c]	% ident[a]
Spo100	490 & 1194 bp	*A.lactucae*	**KY126156**	JVPA01 [T] y ANC4052	97.7	*A.lactucae*	KY126185	LUH 10243	100	*A.lactucae*	KY126219	LUH 7351	99.8
Spo101	*A. baumannii*	*A. baumannii*	**KY126107**	CIP 70.10	99.4	*A. baumannii*	KY126158	CIP 70.10	100	*A. baumannii*	KY126187	CIP 70.10	100

[a] Minimum identity of 99% and of 94% with type or reference strains was considered necessary for reliable species-identification by *gyr*B- and *rpo*B1/*rpo*B2-sequencing, respectively [16, 17].

[b] GenBank accession numbers assigned in this study. New sequences are in bold.

[c] Type and reference strains used in this study:

- *A. baumannii*: CIP 70.34 [T], CIP 70.10, CIP 70.22, ATCC 17904, ATCC 17978, LUH 4708, LUH 5875, NIPH 67, NIPH 335, NIPH 601, NIPH 1734, ACICU, and SDF.
- *A. calcoaceticus*: CIP 81.08 [T], ANC 41.04, ANC 3811, ANC 4221.
- *A. pittii*: CIP 70.29 [T], CIP 70.15, LMG 10560, LMG 10562, LMG 10559, ANC 4626, ANC 5638, NIPH 2805, and PHEA-2.
- *A. nosocomialis*: CIP 70.11 [T].
- *A. seifertii*: CIP 110471 [T].
- *A. lactucae/dijkshoorniae* [5]: JVAP01 [T], LUH 7351, LUH 10243, LUH 13626, ANC 4052.
- *A. courvalinii* (gn. sp. 14BJ): ANC 3623 [T], ANC 4230, ANC 4930, NIPH 1850.
- *A. haemolyticus*: CIP 64.3 [T].
- *A. lwoffii*: CIP 64.10 [T].
- *A. proteolyticus* (gn. sp. 19): NIPH 809 [T], ANC 3839.
- *A. schindleri*: CIP 107287 [T] (ATCC BAA-618 [T]), ACE.

CIP: Collection de l'Institut Pasteur, Paris, France.
ATCC: American Type Culture Collection, Massanas, VA, USA.
LMG: Bacteria Collection, Laboratorium voor Microbiologie en Microbiele Genetica, Gent, Belgium.
LUH and RUH: Collection L. Dijkshoorn, Leiden University Medical Center, Leiden, The Netherlands.
ANC and NIPH: A. Nemec Collection, National Institute of Public Health, Prague, Czech Republic.
DSM and DSMZ: Deutsche Sammlung von Mikroorganismen und Zellkulturen GmbH, Braunschweig, Germany.

[d] Abbreviations: NA, not available; not applicable analysis are indicated with a dash.

References

1. Higgins PG, Wisplinghoff H, Krut O, Seifert H. (2007). A PCR-based method to differentiate between *Acinetobacter baumannii* and *Acinetobacter* genomic species 13TU. Clin Microbiol Infect. 13, 1199-1222.
2. Higgins PG, Lehmann M, Wisplinghoff H, Seifert H. (2010). gyrB multiplex PCR to differentiate between *Acinetobacter calcoaceticus* and *Acinetobacter* genomic species 3. J Clin Microbiol. 48, 4592-4594.
3. Clinical and Laboratory Standards Institute. *Interpretive Criteria for Identification of Bacteria and Fungi by DNA Target Sequencing; Approved Guideline*. CLSI document MM18-A. CLSI 2008, Wayne, PA, USA.

4. La Scola B, Gundi VA, Khamis A, Raoult D. (2006). Sequencing of the *rpo*B gene and flanking spacers for molecular identification of *Acinetobacter* species. J Clin Microbiol. 44, 827-832.
5. Dunlap CA, Rooney AP. (2018). *Acinetobacter dijkshoorniae* is a later herotypic synonym of *Acinetobacter lactucae*. Int J Syst Evol Bacteriol. 68, 131-132.
