# Supplementary material for: Dynamics of a Sporadic Nosocomial Acinetobacter calcoaceticus – Acinetobacter baumannii Complex Population
Source: Front Microbiol. 2019 Mar 22;10:593. doi: 10.3389/fmicb.2019.00593 (PMC6440288; doi:10.3389/fmicb.2019.00593)
Supplement: Supplementary file 2 [file Data_Sheet_2.PDF]

Supplementary Table 2. Sporadic (Spo) and endemoepidemic (EE) *Acinetobacter* spp. PFGE types: epidemiological data, identification of species, OXA-51 allelic variants and sequence types

PFGE type	No. isolates[a]			Year	Ward[a]	Epidemiological status[b]	*Acinetobacter* species	OXA-51 allele[a, c]	ST[a,c]
	Clinic	Environ	NI						
EE1	12	9	0	1999-2001	3F	Epidemic-Outbreak1	*A. baumannii*	OXA-66	2
EE2	11	8	0	2001-2003	2F, 4F, SR, ICU	Endemic	*A. baumannii*	OXA-66	2
EE3	0	1	0	2002	4F	Endemic	*A. baumannii*	OXA-66	2
EE4	6	7	0	2000-2001	3F, ICU	Endemic	*A. baumannii*	OXA-66	2
EE5	6	4	0	2002	3F	Endemic	*A. baumannii*	OXA-66	2
EE6	7	0	0	2005-2006	3F, OC	Endemic	*A. baumannii*	OXA-66	2
EE7	3	3	0	2002	3F, ICU	Endemic	*A. baumannii*	OXA-66	2
EE8	11	4	0	2001-2003	3F, 4F, ICU, ER, OC	Endemic	*A. baumannii*	OXA-66	2
EE9	9	0	1	2005-2008	3F, 4F, 5F	Endemic	*A. baumannii*	OXA-338	80
EE10	11	2	0	2009-2010	ICU	Epidemic-Outbreak3	*A. baumannii*	OXA-66	2
EE11	33	5	0	2003-2007, 2009	3F, 4F, 5F, ICU, ER, OC	Endemic	*A. baumannii*	**OXA-555**	15
EE12	1	0	0	2008	3F	Endemic	*A. baumannii*	OXA-71	3
EE13	58	0	0	2005-2009	3F, 4F, 5F, ICU, ER, OC	Epidemic-Outbreak2	*A. baumannii*	OXA-71	3
EE14	9	0	0	2005, 2007	3F, 4F, 5F, OC, NI	Endemic	*A. baumannii*	OXA-71	3
EE15	4	8	0	2000-2001	3F	Epidemic-Outbreak1	*A. baumannii*	OXA-71	3
Spo1	2	0	0	1999	3F	Sporadic	*A. baumannii*	OXA-66	2
Spo2	3	0	0	1999	3F	Sporadic	*A. baumannii*	OXA-66	2
Spo3	1	0	0	1999	3F	Sporadic	*A. baumannii*	OXA-66	2
Spo4	1	0	0	2000	3F	Sporadic	*A. baumannii*	OXA-66	2
Spo5	2	1	0	2000	3F	Sporadic	*A. baumannii*	OXA-66	2
Spo6	1	0	0	2000	3F	Sporadic	*A. baumannii*	OXA-120	132
Spo7	2	0	0	2000	3F	Sporadic	*A. baumannii*	OXA-66	2
Spo8	1	0	0	2000	3F	Sporadic	*A. baumannii*	**OXA-554**	**687**
Spo9	1	0	0	2000	3F	Sporadic	*A. baumannii*	OXA-66	2
Spo10	0	1	0	2000	3F	Sporadic	*A. baumannii*	OXA-33	54
Spo11	1	0	0	2000	NI	Sporadic	*A. baumannii*	OXA-66	2
Spo12	0	1	0	2000	3F	Sporadic	*A. schindleri*	ND	NA
Spo13	0	2	0	2000	3F	Sporadic	*A. baumannii*	OXA-66	2

PFGE type	No. isolates[a]			Year	Ward[a]	Epidemiological status[b]	*Acinetobacter* species	OXA-51 allele[a, c]	ST[a,c]
	Clinic	Environ	NI						
Spo14	1	0	0	2000	3F	Sporadic	*A. baumannii*	OXA-78	**849**
Spo15	1	0	0	2000	3F	Sporadic	*A. baumannii*	OXA-120	**688**
Spo16	0	0	1	2001	4F	Sporadic	*A. haemolyticus*	ND	NA
Spo17	1	0	0	2001	4F	Sporadic	*A. baumannii*	OXA-93	**689**
Spo18	1	0	0	2001	ICU	Sporadic	*A. baumannii*	**OXA-555**	239
Spo19	2	0	0	2002	3F	Sporadic	*A. baumannii*	OXA-66	2
Spo20	6	0	0	2002-2004	3F, 4F, 5F, ICU, ER	Sporadic	*A. baumannii*	OXA-120	132
Spo21	1	0	0	2002	3F	Sporadic	*A. baumannii*	OXA-120	132
Spo22	1	0	0	2002	3F	Sporadic	*A. baumannii*	OXA-120	193
Spo23	1	0	0	2002	3F	Sporadic	*A. baumannii*	NA	NA
Spo24	1	0	0	2002	4F	Sporadic	*A. baumannii*	OXA-374	**690**
Spo25	0	1	0	2002	3F	Sporadic	*A. pittii*	ND	207
Spo26	1	0	0	2002	3F	Sporadic	*A. pittii*	ND	667
Spo27	0	1	0	2002	4F	Sporadic	*A. pittii*	ND	**691**
Spo28	1	0	0	2002	4F	Sporadic	*A. pittii*	ND	**850**
Spo29	0	1	0	2002	3F	Sporadic	*A. pittii*	ND	64
Spo30	1	0	0	2002	5F	Sporadic	*A. dijkshoorniae*	ND	797
Spo31	1	0	0	2003	3F	Sporadic	*A. baumannii*	OXA-117	139
Spo32	1	0	0	2003	4F	Sporadic	*A. baumannii*	OXA-68	10
Spo33	1	0	0	2003	5F	Sporadic	*A. baumannii*	OXA-120	**692**
Spo34	1	0	0	2003	ICU	Sporadic	*A. baumannii*	OXA-208	**851**
Spo35	1	0	0	2003	ICU	Sporadic	*A. pittii*	ND	**852**
Spo36	1	0	0	2003	NI	Sporadic	*A. pittii*	ND	**853**
Spo37	0	0	2	2003	3F, 5F	Sporadic	*A. dijkshoorniae*	ND	**854**
Spo38	1	0	0	2003	3F	Sporadic	*A. pittii*	ND	**855**
Spo39	1	0	0	2004	5F	Sporadic	*A. baumannii*	OXA-66	2
Spo40	1	0	0	2004	3F	Sporadic	*A. baumannii*	OXA-66	2
Spo41	1	0	0	2004	4F	Sporadic	*A. baumannii*	NA	**693**
Spo42	1	0	0	2004	OC	Sporadic	*A. pittii*	ND	**856**
Spo43	1	0	0	2004	4F	Sporadic	*A. baumannii*	OXA-120	132

PFGE type	No. isolates[a]			Year	Ward[a]	Epidemiological status[b]	*Acinetobacter* species	OXA-51 allele[a, c]	ST[a,c]
	Clinic	Environ	NI						
Spo44	2	0	0	2004	4F, ER	Sporadic	*A. baumannii*	OXA-126	**857**
Spo45	1	0	0	2004	OC	Sporadic	*A. pittii*	ND	214
Spo46	1	0	0	2004	4F	Sporadic	*A. baumannii*	**OXA-556**	**694**
Spo47	2	0	0	2004	ICU	Sporadic	*A. baumannii*	**OXA-557**	464
Spo48	1	0	0	2004	1F	Sporadic	*A. baumannii*	OXA-120	448
Spo49	2	0	0	2004	3F	Sporadic	*A. baumannii*	OXA-66	2
Spo50	1	0	0	2004	5F	Sporadic	*A. pittii*	ND	64
Spo51	2	0	0	2004-2005	4F, ICU	Sporadic	*A. pittii*	ND	**832**
Spo52	1	0	0	2004	ER	Sporadic	*A. baumannii*	OXA-66	2
Spo53	1	0	0	2004	3F	Sporadic	*A. baumannii*	OXA-120	132
Spo54	1	0	0	2004	4F	Sporadic	*A. baumannii*	**OXA-555**	**858**
Spo55	2	0	0	2004-2005	3F, ICU	Sporadic	*A. baumannii*	OXA-120	132
Spo56	1	0	0	2005	3F	Sporadic	*A. pittii*	ND	NA
Spo57	1	0	0	2005	OC	Sporadic	*A. baumannii*	**OXA-558**	**859**
Spo58	1	0	0	2005	OC	Sporadic	*A. baumannii*	OXA-338	80
Spo59	2	0	0	2005-2006	5F	Sporadic	*A. baumannii*	**OXA-555**	**695**
Spo60	2	0	0	2005-2006	3F, 4F	Sporadic	*A. calcoaceticus*	ND	**860**
Spo61	2	0	0	2006	3F, OC	Sporadic	*A. proteolyticus*	ND	NA
Spo62	1	0	0	2006	3F	Sporadic	*A. dijkshoorniae*	ND	**861**
Spo63	1	0	0	2006	NI	Sporadic	*A. baumannii*	**OXA-558**	**862**
Spo64	1	0	0	2006	OC	Sporadic	*A. pittii*	ND	249
Spo65	1	0	0	2006	3F	Sporadic	*A. pittii*	ND	NA
Spo66	1	0	0	2006	OC	Sporadic	*A. dijkshoorniae*	ND	**863**
Spo67	1	0	0	2006	3F	Sporadic	*A. baumannii*	OXA-66	2
Spo68	1	0	0	2006	OC	Sporadic	*A. pittii*	ND	**864**
Spo69	1	0	0	2007	3F	Sporadic	*A. pittii*	ND	**865**
Spo70	2	0	0	2007	3F, 4F	Sporadic	*A. baumannii*	OXA-66	2
Spo71	3	0	0	2007	3F, 5F, ICU	Sporadic	*A. baumannii*	**OXA-555**	15
Spo72	3	2	0	2007	ICU, OC	Sporadic	*A. baumannii*	OXA-385	**866**
Spo73	1	0	0	2007	OC	Sporadic	*A. baumannii*	**OXA-559**	**696**

PFGE type	No. isolates[a]			Year	Ward[a]	Epidemiological status[b]	*Acinetobacter* species	OXA-51 allele[a, c]	ST[a,c]
	Clinic	Environ	NI						
Spo74	1	0	0	2007	3F	Sporadic	*A. baumannii*	OXA-66	2
Spo75	1	0	0	2007	4F	Sporadic	*A. courvalinii*	ND	NA
Spo76	1	0	0	2007	5F	Sporadic	*A. calcoaceticus*	ND	**956**
Spo77	1	0	0	2007	4F	Sporadic	*A. baumannii*	OXA-100	32
Spo78	1	0	0	2007	ICU	Sporadic	*A. baumannii*	OXA-66	2
Spo79	1	0	0	2008	OC	Sporadic	*A. baumannii*	OXA-66	2
Spo80	2	0	0	2007-2008	3F, ER	Sporadic	*A. baumannii*	**OXA-560**	285
Spo81	1	0	0	2008	3F	Sporadic	*A. baumannii*	**OXA-555**	**867**
Spo82	1	0	0	2008	3F	Sporadic	*A. baumannii*	OXA-69	153
Spo83	1	0	0	2008	5F	Sporadic	*A. baumannii*	OXA-120	132
Spo84	2	0	0	2008	3F	Sporadic	*A. baumannii*	**OXA-555**	239
Spo85	1	0	0	2008	1F	Sporadic	*A. baumannii*	**OXA-561**	250
Spo86	1	0	0	2008	5F	Sporadic	*A. baumannii*	OXA-71	3
Spo87	2	0	0	2008-2009	5F, ER	Sporadic	*A. baumannii*	OXA-66	**697**
Spo88	1	0	0	2008	5F	Sporadic	*A. pittii*	ND	**868**
Spo89	1	0	0	2008	3F	Sporadic	*A. pittii*	ND	**833**
Spo90	1	0	0	2008	4F	Sporadic	*A. baumannii*	**OXA-562**	370
Spo91	1	0	0	2009	4F	Sporadic	*A. baumannii*	OXA-69	153
Spo92	1	0	0	2009	ER	Sporadic	*A. baumannii*	OXA-104	**869**
Spo93	1	0	0	2009	4F	Sporadic	*A. dijkshoorniae*	ND	**870**
Spo94	1	0	0	2009	4F	Sporadic	*A. baumannii*	**OXA-555**	15
Spo95	1	0	0	2009	OC	Sporadic	*A. pittii*	ND	**871**
Spo96	1	0	0	2009	OC	Sporadic	*A. pittii*	ND	93
Spo97	1	0	0	2009	4F	Sporadic	*A. pittii*	ND	64
Spo98	1	0	0	2009	3F	Sporadic	*A. baumannii*	OXA-71	3
Spo99	1	0	0	2009	ER	Sporadic	*A. baumannii*	OXA-95	332
Spo100	1	0	0	2009	OC	Sporadic	*A. dijkshoorniae*	ND	**872**
Spo101	1	0	0	2009	3F	Sporadic	*A. baumannii*	OXA-120	132

[a]Abbreviations: Clinic, clinical; Environ, environmental; NI, not informed; 1F, first floor; 2F, second floor; 3F, third floor; 4F, fourth floor; 5F, fifth floor; ICU, intensive care unit; SR, surgery room; ER, emergency room; OC, out-patient clinic consultation room; ND, not detected; NA, not assigned.

[b]Three *A. baumannii* nosocomial outbreaks occurred in the studied period: outbreak1 in 1999-2000, outbreak2 in 2006-2007, and outbreak3 in 2009-2010 [13].
[c]New OXA-51 alelles and new STs described in this work are in bold.
